# Supplementary material for: Interpretable machine learning prediction of all-cause mortality
Source: Commun Med (Lond). 2022 Oct 3;2:125. doi: 10.1038/s43856-022-00180-x (PMC9530124; doi:10.1038/s43856-022-00180-x)
Supplement: Supplementary file 1 — Supplementary Information [file 43856_2022_180_MOESM1_ESM.pdf]

Interpretable machine learning prediction of all-cause mortality
Supplementary Information

Wei Qiu[1], Hugh Chen[1], Ayse Berceste Dincer[1], Scott Lundberg[2], Matt Kaeberlein[3], and Su-In Lee[1,*]

[1]Paul G. Allen School of Computer Science and Engineering, University of Washington, Seattle, WA, USA
[2]Microsoft Research, Redmond, WA, USA
[3]Department of Laboratory Medicine and Pathology, University of Washington, Seattle, WA, USA
[*]Corresponding: suinlee@cs.washington.edu

Supplementary Methods

Data collection and processing

The National Health and Nutrition Examination Survey (NHANES) from the National Center for Health Statistics (NCHS)(`http://www.cdc.gov/nchs/nhanes.htm`) conducts interviews and physical examinations to assess the health and nutrition data for all ages in the United States. The interviews include demographic, socioeconomic, dietary, and health-related questions. The examinations include medical, dental, physiological measurements, and laboratory tests administered by highly trained medical personnel. Since 1999, data were collected and released at 2-year intervals. Each year NHANES examines a nationally representative sample of roughly 5,000 individuals across the Unites States. The design of the sample changed periodically. Oversampled subgroups for 1999–2006 included non-Hispanic black persons, Mexican-American persons, low-income white persons (beginning in 2000), adolescents aged 12–19, and persons aged 70 and over. Oversampled subgroups for 2007–2010 included all Hispanic persons, non-Hispanic black persons, low-income white persons, and persons aged 80 and over (`https://www.cdc.gov/nchs/data/series/sr\_01/sr01\_056.pdf`). In this study, we include NHANES data sampled between 1999 and 2014. All-cause mortality is ascertained by a linked NHANES mortality file that provides follow-up mortality data from the date of survey participation through December 31, 2015. We exclude participants under age 18 because they are not eligible for public release mortality data (`https://www.cdc.gov/nchs/data/datalinkage/public-use-2015-linked-mortality-file-description.pdf`).

Our study includes samples with known mortality status who participated in NHANES 1999-2014 ($n = 47,261$). In the raw data, individuals 85 and over are topcoded at 85 years of age in NHANES 1999-2006 and individuals 80 and over are topcoded at 80 years of age in NHANES 2007-2014. To keep consistency, we topcode individuals 80 and over at 80 years of age. The histogram of the the samples' age in different data collection cycles are shown in Supplementary Figure 2. We include all demographic, laboratory, examination, and questionnaire features that could be automatically matched across different NHANES cycles. We exclude variables that are missing for more than 50% of the participants and highly correlated features with correlations greater than 0.98; after filtering and one-hot encoding, 151 features remain (Supplementary Data 1). We impute missing data using MissForest[8], a nonparametric random forest-based multiple imputation method for mixed-type data, with seven iterations. We predict all-cause mortality for two broad categories: (1) follow-up times of 1-year, 3-year, and 5-year and (2) age groups of $<$40, 40-65, 65-80, and $\geq$80 years old. For different follow-up times, we remove samples with unconfirmed mortality status. For different age groups, we predict 5-year mortality. The demographic characteristics and sample size of the data for different tasks are shown in Supplementary Table 1.

We use UK Biobank samples as an external validation dataset. Participants were enrolled in the UK Biobank from April, 2007, to July, 2010, from 21 assessment centres across England, Wales, and Scotland using standardised procedures. When participants agreed to take part in UK Biobank, they visited their closest assessment centre to provide baseline information, physical measures, and biological samples. We include the 51 features that are overlapping between NHANES and UK Biobank dataset (Supplementary Data 1). We exclude samples with missing values. All-cause mortality included all deaths occurring before May, 2021. We include 384,762 samples aged 37-72 years with confirmed 5-year mortality status. Of these samples, 6,336 died after 5 years. The histograms of age, gender and body mass index of UK Biobank samples are shown in Supplementary Figure 3.

Predictive modeling

To model mortality, we use gradient boosted trees (GBTs). GBTs are nonparametric methods composed of iteratively trained decision trees. The final ensemble of trees captures non-linearity and interactions between predictors. The dataset is randomly divided into training (80%) and testing (20%) sets. We use the implementation XGBoost[1] (`https://xgboost.readthedocs.io/en/latest/python/index.html`) with a learning rate set to 0.002 , subsample ratio set to 0.5 and 10,000 trees of max depth 3. For comparison, we also train logistic regression models and deep neural networks. For logistic regression, we use L2 regularization. The L2 regularization weight was set to 100. For neural networks, we use a single layer with 1,000 nodes, and max iteration set to 1,000. The hyperparameters specified above are chosen by GridSearch and 5-fold cross validation. Other hyperparameter values are left at their default values. Models' performance is measured with the area under the receiver operator characteristic curve (AUROC) and the area under the precision-recall curve (AUPRC). We bootstrap the test set for 1,000 times to assess the statistical significance of the difference in AUROC and AUPRC for pairs of models. Specifically, we resample with replacement from the test set 1,000 times and compare the models' performance on resampled test sets. We report a p-value which is the percentage of time that logistic regression or the neural network's performance is better than or equal to gradient boosted trees, divided by the number of resampled test sets. All models are built using the Scikit-learn package in Python 3.7.

Model interpretation

To explain the GBT models, we utilize TreeExplainer[7], which provides a local explanation of the impact of input features on individual predictions. Specifically, TreeExplainer calculates exact SHAP[6] (SHapley Additive exPlanations) values for tree-based models. When explaining the mortality prediction models, we randomly select 10,000 background samples from the training set and 5,000 foreground samples from the test set.

SHAP (SHapley Additive exPlanation) values

SHAP (SHapley Additive exPlanation) values attribute to each feature the change in the expected model prediction when conditioning on that feature. The change of the model's prediction when the feature is masked is recorded across all possible subsets of features, yielding an average change in prediction resulting from the inclusion of a feature in the model:

$$\phi_i(f,x) = \sum_{R\in\mathcal{R}} \frac{1}{M!} \left[f_x(P_i^R \cup i) - f_x(P_i^R) \right], \tag{3}$$

where ϕ_i is the feature attribution (SHAP value) of feature i in model f for data point x, $\mathcal{R}$ is the set of all feature permutations, P_i^R is the set of all features before i in the ordering R, M is the number of input features, and f_x is an estimate of the conditional expectation of the model's prediction: $f_x(S) \approx \mathrm{E[f(x) \mid x_S]}$ where x_S is the set of observed features.

SHAP values which guarantee a set of desirable theoretical properties, including additivity and consistency. Additivity states that when approximating the original model f for a specific input x, the SHAP values sum up to the output $f(x)$:

$$f(x) = \phi_0(f) + \sum_{i=1}^{M} \phi_i(f, x), \tag{4}$$

The sum of feature attributions (SHAP values) matches the original model output f(x), where $\phi_0(f) = \mathrm{E[f(z)] = f_x(\emptyset)}$. Consistency states that if a model changes so that some feature's contribution increases or stays the same regardless of the other inputs, that input's attribution should not decrease. Therefore, SHAP values are consistent and accurate calculations of each feature's contribution to the model's prediction.

SHAP interaction values and main effects

The SHAP interaction effects is based on the Shapley interaction index from game theory. While standard feature attribution results in a vector of values, one for each feature, attributions based on the Shapley interaction index result in a matrix of feature attributions. The main effects are on the diagonal and the interaction effects on the off-diagonal. The **SHAP interaction values** are defined as:

$$\Phi_{i,j}(f, x) = \sum_{S \subseteq \mathcal{M} \setminus \{i,j\}} \frac{|S|!(M - |S| - 2)!}{2(M-1)!} \nabla_{i,j}(f, x, S), \tag{5}$$

when $i \neq j$, and

$$\nabla_{ij}(f, x, S) = f_x(S \cup \{i, j\}) - f_x(S \cup \{i\}) - f_x(S \cup \{j\}) + f_x(S). \tag{6}$$

where $\mathcal{M}$ is the set of all M input features. In Equation 5 the SHAP interaction value between feature i and feature j is split equally between each feature so $\Phi_{i,f}(f, x) = \Phi_{j,i}(f, x)$ and the total interaction effect is $\Phi_{i,f}(f, x) + \Phi_{j,i}(f, x)$.

The **main effects** for a prediction can then be defined as the difference between the SHAP values and the off-diagonal SHAP interaction values for a feature:

$$\Phi_{i,i}(f, x) = \phi_i(f, x) - \sum_{j \neq i} \Phi_{i,j}(f, x). \tag{7}$$

*Partial dependence plots and additional perspective to reference interval We use partial dependence plots to show the change in mortality risk for all values of a laboratory feature. Partial dependence plots show the marginal effect a set of features has on the prediction of an ML model. The partial function f_S is estimated by:

$$f_S(x_S) = \frac{1}{n} \sum_{i=1}^{n} f(x_S, x_C^{(i)}). \tag{8}$$

In this formula, f is a ML model and the S are features for which the partial dependence function should be plotted. In our study, S is the laboratory feature of interest and x_S is the given value of the feature. $x_C^{(i)}$

is actual feature values for the features of no interest in the test set, and n is the number of instances in the test set. The partial function tells us the average marginal effect on the prediction for given value(s) of features S. We extend the partial function to the relative mortality risk RR_S:

$$RR_S(x_S) = f_S(x_S)/(\frac{1}{n}\sum_{i=1}^{n} f(x^{(i)})). \tag{9}$$

In other words, the relative mortality risk is defined as the average value of the model's predicted probability when we fix a specific feature to a given value divided by the average value of the model's predicted probability. We further define the relative risk percentage (RRP) as follows:

$$RRP_S = \frac{max(RR_S(x_S), x_S \ in \ RI) - 1}{max(RR_S(x_S)) - 1}, \tag{10}$$

where RI stands for reference interval. High relative risk percentage indicates that the values within the reference interval have a relatively high mortality risk. The partial dependence plots of selected laboratory feature values on 1-, 3-, and 10-year mortality risk are shown in Supplementary Figure 5.

Model interpretation plots

In this section we describe a number of plotting types for model explanation visualization.

SHAP value, SHAP main effect value and SHAP interaction value plots In SHAP value/SHAP main effect value/SHAP interaction value plots, every point corresponds to a single sample where the x-axis is the value of the feature and the y-axis is the SHAP value/SHAP main effect value/SHAP interaction value. The coloring of the points often denotes the value of a separate feature.

Summary plot Summary plots show the feature attributions (SHAP values) for many samples and multiple features in order of global feature importance (the mean absolute SHAP values). Summary plots stack multiple subplots for each feature. For the feature plots, every point corresponds to a single sample where the x-axis is the feature attribution value and the y-axis is vertical dispersion representing the frequency of samples with a particular feature attribution value. Finally, the color of each point represents the normalized feature value, with red representing a high value and blue representing a low one. Intermediary feature values are interpolations between red and blue.

Individualized explanation plot Individualized explanation plots show the feature attributions (SHAP values) for an individual in terms of how they drive the model's prediction for the individual away from the average model prediction across the baseline distribution. The width of the bars indicate the SHAP value with red indicating a positive affect and blue indicating a negative one. The features corresponding to the largest bars are below with their actual values for the individual.

Supervised distance

Supervised distance and hierarchical clustering

Supervised distance can accurately measure feature redundancy based on a specific prediction task. As Supplementary Figure 11 shows, to calculate the supervised distance between feature i and feature j, we

first train a uni-variate GBT model to predict the label (e.g. 5-year mortality in our study) using feature i. Then, we can obtain the $Prediction_i$ which is the output of the fitted uni-variate GBT. Next, we fit another uni-variate GBT to predict $Prediction_i$ using feature j. We define the output of the new GBT as $Prediction_i^j$. All hyperparameter values of the uni-variate GBTs are set to their default values. Following the same above steps, we can obtain $Prediction_j^i$. The supervised distance between feature i and feature j (supervised distance(i,j)) is defined as:

$$supervised\,R^2(i,j) = max(0, 1 - mean(\frac{(Prediction_i - Prediction_i^j)^2}{var(Prediction_i)})) \tag{11}$$

$$supervised\,distance(i,j) = max(1 - supervised\,R^2(i,j), 1 - supervised\,R^2(j,i)) \tag{12}$$

where $var(x)$ is the variance of the vector x, $mean(x)$ is the average of the vector x. Supervised distance is scaled roughly between 0 and 1, where 0 distance means the features perfectly redundant and 1 means they are completely independent.

To explore the redundant feature groups, we hierarchically cluster all features according to the supervised distance. Specifically, we use complete linkage hierarchical clustering which merges in each step the two clusters whose merger has the smallest diameter. The hierarchical clustering tree is shown in Supplementary Figure 12.

Redundant feature groups experiments training details

Reducing redundancy model To identify the most representative feature in a redundant feature group, we train GBTs using one feature in the redundancy group and all features outside the group for 5-year mortality prediction. Then we compare the feature importance ranking of the redundant features by calculating the mean absolute SHAP values using TreeExplainer. The hyperparameters of the GBTs are chosen by GridSearch and 5-fold cross validation. The max depth is selected from $\{1, 3, 5, 7, 9\}$ and the subsample ratio is selected from $\{0.2, 0.5, 0.8, 1.0\}$. Other hyperparameter values are left at their default values.

Single feature model We further analyze the predictive power of the redundant features by fitting 5-year mortality prediction GBTs using one feature in the redundant feature group. Specifically, we use one feature in the redundant feature group and two important confounders, age and gender, to train a GBTs for 5-year mortality prediction. All hyperparameter values are set to their default values. We compare the AUCs of the models. We bootstrap the test set for 1,000 times and compare the models' performance on resampled test sets. The averages of the AUCs are reported.

Supervised distance-based feature selection

We propose a supervised distance-based feature selection method to select predictive and less-redundant feature sets. The workflow of our feature selection method is shown in Supplementary Figure 11. The dataset is randomly divided into training (80%) and testing (20%) sets. Firstly, we fit a GBT for 5-year mortality prediction on all features using the training set and rank the features by mean absolute SHAP values from TreeExplainer. The hyperparameters of the GBTs are chosen by GridSearch and 5-fold cross validation. The max depth is selected from $\{1, 3, 5, 7, 9\}$ and the subsample ratio is selected from $\{0.2, 0.5, 0.8, 1.0\}$. The max number of trees is set to 1000. We use 20% of the training samples as validation set for early stopping. The number of early stopping rounds is set to 100. Since age and gender are important confounders, we

would like to keep them in the selected feature set. Therefore, we cluster features except age and gender into a specific number of groups using supervised distances-based hierarchical clustering and select the most important feature in each cluster. Then, we add age and gender to the selected feature set and re-fit the model. Next, we rerun the clustering using the new feature set except age and gender. This process is repeated until all remaining features cluster to a single group. In every iteration, we remove 5 features. The models are evaluated on the testing set with bootstrapping for 1,000 times. We report the average of the AUROCs and the minimum supervised distance within the selected feature sets. The selected features in each iteration are listed in Supplementary Data 1.

5-year mortality risk scores

Mortality risk scores training details

IMPACT mortality risk scores are defined to be the prediction of the 5-year mortality prediction models. For comparison, we train linear (`https://scikit-survival.readthedocs.io/en/latest/api/generated/sksurv.linear\_model.CoxPHSurvivalAnalysis.html`) and gradient boosted tree-based Cox proportional hazard models (`https://scikit-survival.readthedocs.io/en/latest/api/generated/sksurv.ensemble.GradientBoostingSurvivalAnalysis.html`). We do a temporal validation of the risk scores by assessing their performances in the samples collected in 2009-2014 ($N = 7,034$). Specifically, the samples collected in 1999-2008 ($N = 28,820$) are randomly divided into training (80%) and testing (20%) sets. The sample size, the number of deceased samples and the histogram of age in the training set with the testing set and the temporal validation set are shown in Supplementary Figure 13. To compare with Intermountain gender-specific risk scores, we evaluate the models on different gender groups. The models are trained on the whole training set and evaluate on different gender groups in the testing set. Furthermore, considering the different feature collection cost for the general public and medical professionals, we build the risk scores starting from different feature sets. For the general public, the models are trained on all demographics, questionnaire features and examination features that are accessible at home for general public, For medical professionals, the models are trained on all demographics and laboratory features. All trained models are evaluated on different gender groups of the samples collected in 2009-2014 for temporal validation.

The hyperparameters are chosen by GridSearch and 5-fold cross validation. For XGBoost 5-year mortality prediction models, the max depth is selected from $\{1, 3, 5, 7, 9\}$ and the subsample ratio is selected from $\{0.2, 0.5, 0.8, 1.0\}$. The max number of trees is set to 1000. We use 20% of the training samples as validation set for early stopping. The number of early stopping rounds is set to 100. For linear Cox proportional hazard models, the regularization parameter α is selected from $\{0.01, 0.1, 1, 10, 100\}$. For tree-based Cox proportional hazard models, the max depth is selected from $\{1, 3, 5, 7, 9\}$ and the subsample ratio is selected from $\{0.2, 0.5, 0.8, 1.0\}$. Other hyperparameter values are left at their default values.

We explain the mortality prediction model in terms of its probability predictions. Specifically, we rescale the SHAP values (in the log-odds space) to be in the probability space directly. The rescaled SHAP values now sum to the probability output of the model.

Recursive feature elimination

Recursive feature elimination works by searching for a subset of features by starting with all features in the training dataset and successively removing features until the desired number of features remains. Firstly, we train a model on the full dataset with all features. Then we rank features by importance (mean absolute

SHAP values) and remove the least important features. Another model is trained on the resulting feature set, and the process iterates until only the desired number of features are left. Starting from 151 features, we remove 6 features at the first iteration. Then, we remove 5 features in each iteration until only one feature is left. We bootstrap the test set for 1,000 times and assess the predictive performance. Specifically, we resample with replacement from the test set 1,000 times and report the average and the 95% confidence interval of the AUROCs. The selected features in each iteration are listed in Supplementary Data 1.

Intermountain mortality risk score

Intermountain mortality risk scores[4] are built using complete blood count and basic metabolic profile. Specifically, 13 laboratory features are used to predict 30 days, 1-year and 5-year mortality. Logistic regression was used to model the risk prediction equations with adjustment for age and sex. Dummy variables modeled each category, with the referent defined as the lowest risk group (except for age categories: 18-29, 30-39, 40-49 [referent], 50-59, 60-69, 70-79, and $\geq$80 years). A scalar score value was derived for each variable category by multiplying its β-coefficient by 3 and rounding to the nearest integer (referent value = zero). Each individual's risk score became the sum of the score values based on his or her individual data. Since all of the features used in the Intermountain risk scores are included in our NHANES dataset, we evaluate the Intermountain risk score on our NHANES testing set with bootstrapping for 1,000 times.

Comparing the predictive power of popular mortality risk scores and biological ages with IMPACT

Since not all features used in the popular mortality risk scores and biological ages are included in the NHANES dataset (except for Intermountain risk scores; see Supplementary Methods), it would not be fair to compare the existing mortality scores and biological ages computed based on a partial set of features with the IMPACT model based on the NHANES dataset. Therefore, we chose to show the AUROCs reported in the original papers. As the AUROCs are not sensitive to the base rate, we assume that these scores would be consistent among different datasets if the risk scores and biological ages generalize well.

Table 1 compares the AUROCs between an existing mortality score or a biological age as reported in the original paper and the IMPACT-20 model tested for the corresponding follow-up time and age ranges in the NHANES dataset. Here, IMPACT-20 means the IMPACT model when the top 20 features were used; we chose 20 features because in Figures 8a-b, the IMPACT model with 20 features obtains an AUROC that is almost the same as the performance of the model using all features, and using fewer than 20 features leads to a dramatic decline in accuracy.

To get the top 20 most important features for 1-year and 10-year mortality predictions, we repeat the same mortality risk scores training and recursive feature elimination process for 1-year and 10-year predictions. We perform temporal validation to show the generalizability of the IMPACT-20 risk scores on data collected at different time periods. To have similar base rates and age distributions in the test set and temporal validation set, we use the samples from different collection cycles as the temporal validation set for different follow-up times. For 1-year mortality prediction, we use the samples collected in 1999-2012 as the training/testing set and the samples collected in 2013-2014 as the temporal validation set. For 5-year mortality prediction, we use the samples collected in 1999-2008 as the training/testing set and those collected in 2009-2014 as the temporal validation set. For 10-year mortality prediction, we use the samples collected in 1999-2000 as the training/testing set and those collected in 2001-2014 as the temporal validation set. With respect to the 5-year mortality risk scores, samples that are not included in the temporal validation set are randomly split

into 80% for training and 20% for testing. The sample size, number of deceased samples, and histogram of age in the training set, with the testing and temporal validation sets, are shown in Supplementary Figure 13. In Table 1, the "AUROC" column shows the AUROCs reported in the original paper. The "AUROC of IMPACT-20" column shows the performance of 1-year, 5-year and 10-year IMPACT models trained with the selected top 20 features (listed in Supplementary Tables 2-3). The IMPACT-20 models are trained on samples of all ages and evaluated on the samples within the same age range in the original paper. We bootstrap the test set and the temporal validation set for 1,000 times when measuring the AUROCs.

Supplementary Notes

Supplementary Note 1 External validation of the NHANES mortality prediction model on the UK Biobank (UKB) dataset

We aim to validate whether the performance and explanations of the NHANES mortality prediction model generalize to an unseen population (UKB). To do so, we train a new tree-based 5-year mortality prediction model on the NHANES dataset using the 51 overlapping features between NHANES and UKB. As shown in Supplementary Figure 8h, the classification accuracy on the UKB test set of the model trained on NHANES samples (AUROC = 0.7780) and UKB samples (AUROC = 0.7974) are close, which shows the generalizability of the NHANES model. Supplementary Figure 7a shows the feature importances of the 51 features of the NHANES (51 features) and UKB models. *The SHAP values of both models are calculated using the same UKB samples.* We observe that the top 20 most important features are largely consistent, with 14 features the same for both models. The p-value of the Fisher's exact test (p-value = 0.0004) shows that the overlap between the top 20 most important features of both models is significant. The Spearman's correlation coefficient of both models' feature importance is 0.6969 (p-value < 0.0001). Supplementary Figures 7b-g show noteworthy results of the NHANES (51 features) model explained by UKB samples: the SHAP main effect of red cell distribution width, serum albumin and serum uric acid, and the relative 5-year mortality risk of gamma glutamyl transferase, lymphocyte percent and serum albumin. The trends shown in these figures are consistent with previous findings from both the NHANES (151 features) and UKB (51 features) models. Additional validation results on the UKB dataset are presented in Supplementary Figure 8.

Supplementary Note 2 Discoveries for mortality prediction using different age groups

IMPACT identifies important features for mortality prediction in different age groups.

Supplementary Figure 9a shows the top 20 most important features and relative importance in 5-year mortality prediction models using different age groups (<40, 40-65, 65-80 and ≥80). Some features become more important for older subpopulations, such as alanine aminotransferase (ALT), the fifth most important feature in the model using samples over 80 years. Supplementary Figure 9e shows the main effect of ALT for age≥80, which shows the negative relationship between ALT and 5-year mortality. Moreover, some features are less important for older subpopulations than younger ones. One example is uric acid level, the sixth most important feature in the age<40 model and the 59th most important in the age≥80 model. Supplementary Figure 9b plot the main effect and SHAP value of uric acid in the age<40 model, showing that low uric acid levels increase mortality risk prediction. However, in the age 40-65 model, higher uric acid is associated with higher mortality risk (Supplementary Figure 9d). Previous work shows that low uric acid in blood serum

can injure the endothelium and induce oxidative stress-related disease[9,11], and that hyperuricemia (high uric acid) is associated with various adverse health outcomes, including hypertension, stroke, cardiovascular disease and cancer[2,3,5,10]. The numerous downstream effects of high uric acid and low uric acid might explain the different relationship between uric acid and mortality in different age groups. Moreover, the reference range of uric acid differs for males and females (2.4-6.0 mg/dL for females and 3.4-7.0 mg/dL for males). This difference is shown in Figure 9c, where women have lower uric acid, which can increase mortality risk.

Supplementary Note 3 Explaining the mortality predictions using different baseline distributions

In the Results section, we use TreeExplainer to explain an explicand relative to a baseline distribution drawn uniformly from all training samples (Figure 3a) This explanation substantially emphasizes age because it compares the explicand to the general population baselines that include individuals of all ages. However, in practice, epidemiologists are more interested in an individual's strong risk factors compared with people of the same age. To show this, we can manually select baselines from the samples that have similar age with the explicand. We take the middle-aged (40-50) baseline distribution and the older (60-70) baseline distribution as two examples. Specifically, we use the testing samples in the specific age range as the explicands (i.e., samples being explained) and training samples in the same age range as the baselines (i.e., background samples) when calculating the SHAP values. The SHAP summary plots are shown in Supplementary Figures 10a-b. From the figures, we observe that age is no longer the most important feature. Also, compared with Figure 3a, the SHAP value ranges are relatively similar. Therefore, we can identify the strong mortality predictors other than age for different age groups using different baseline distributions. Supplementary Figures 10c,e show the individualized explanations of a healthier vs unhealthier sample using baselines from the general population. We observe that age contributes a lot to the prediction. However, as shown in Supplementary Figures 10d,f, the contribution of other important risk factors increases when we use older baselines. These examples illustrate that using the baselines with similar age can help identify strong risk factors besides age.

Different follow-up times					
		1-year (n=47,261)	3-year (n=41,434)	5-year (n=35,854)	10-year (n=21,986)
Number of deaths		577 (1.22%)	1,897 (4.58%)	3,074 (8.57%)	5,295 (24.08%)
Age,years		46 (30-63)	46 (30-64)	46 (30-64)	48 (30-67)
Sex					
	Male	22,778 (48.20%)	19,998 (48.26%)	17,276 (48.18%)	10,630 (48.35%)
	Female	24,483 (51.80%)	21,436 (51.74%)	18,578 (51.82%)	11,356 (51.65%)
Ethnicity					
	Mexican American	8,947 (19.93%)	8,164 (19.70%)	7,543 (21.04%)	4,844 (22.03%)
	Other Hispanic	3,452 (7.03%)	2,929 (7.07%)	2,335 (6.51%)	979 (4.45%)
	Non-Hispanic White	21,428 (45.34%)	18,990 (45.83%)	17,081 (47.64%)	10,921 (49.67%)
	Non-Hispanic Black	10,039 (21.24%)	8,821 (21.29%)	7,337 (20.46%)	4,353 (19.78%)
	Other Race	3,395 (7.18%)	2,530 (6.11%)	1,558 (4.35%)	889 (4.04%)
Different age groups (follow-up time = 5-year)					
		Age < 40 (n=14,221)	$40 \leq$ Age < 65 (n=12,705)	$65 \leq$ Age < 80 (n=6,004)	Age ≥ 80 (n=2,924)
Number of deaths		183 (1.29%)	648 (5.10%)	961 (16.01%)	1,282 (43.84%)
Age,years		27 (21-33)	51 (45-58)	71 (68-75)	80 (80-80)
Sex					
	Male	6,629 (46.61%)	6,263 (49.30%)	3,056 (50.90%)	1,328 (45.42%)
	Female	7,592 (53.39%)	6,442 (50.70%)	2,948 (49.10%)	1,596 (54.58%)
Ethnicity					
	Mexican American	3,663 (25.76%)	2,622 (20.64%)	1,046 (17.52%)	212 (7.25%)
	Other Hispanic	993 (6.98%)	906 (7.13%)	329 (5.48%)	107 (3.66%)
	Non-Hispanic White	5,686 (39.98%)	5,825 (45.85%)	3,319 (55.28%)	2,251 (76.98)
	Non-Hispanic Black	3,143 (22.10%)	2,793 (21.98%)	1,125 (18.74%)	276 (9.44)
	Other Race	736 (5.18%)	559 (4.40%)	185 (3.08%)	78 (2.67%)

Supplementary Table 1: Population characteristics for the study cohorts. Data are median (IQR), or n/N (%).

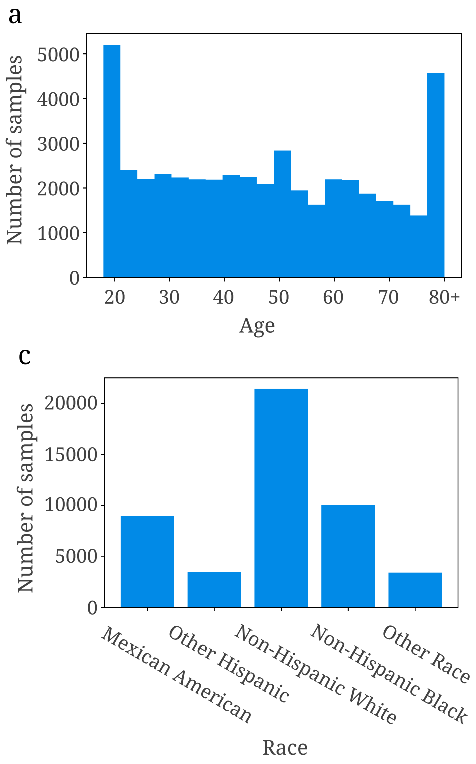

e

	Number of samples	Number of alive samples	Number of deceased samples
1-year mortality	47,261	46,684	577
3-year mortality	41,434	39,537	1,897
5-year mortality	35,854	32,780	3,074
10-year mortality	21,986	5,295	16,691
Age < 40	14,221	14,038	183
40 ≤ Age < 65	12,705	12,057	648
65 ≤ Age < 80	6,004	5,043	961
Age ≥ 80	2,924	1,642	1,282

Supplementary Figure 1: (a–d) Histograms of age, gender, race, and body mass index in the NHANES dataset. (e) The sample size and number of living and deceased samples for different follow-up times and different age groups. For different age groups, the follow-up time is set to 5 years.

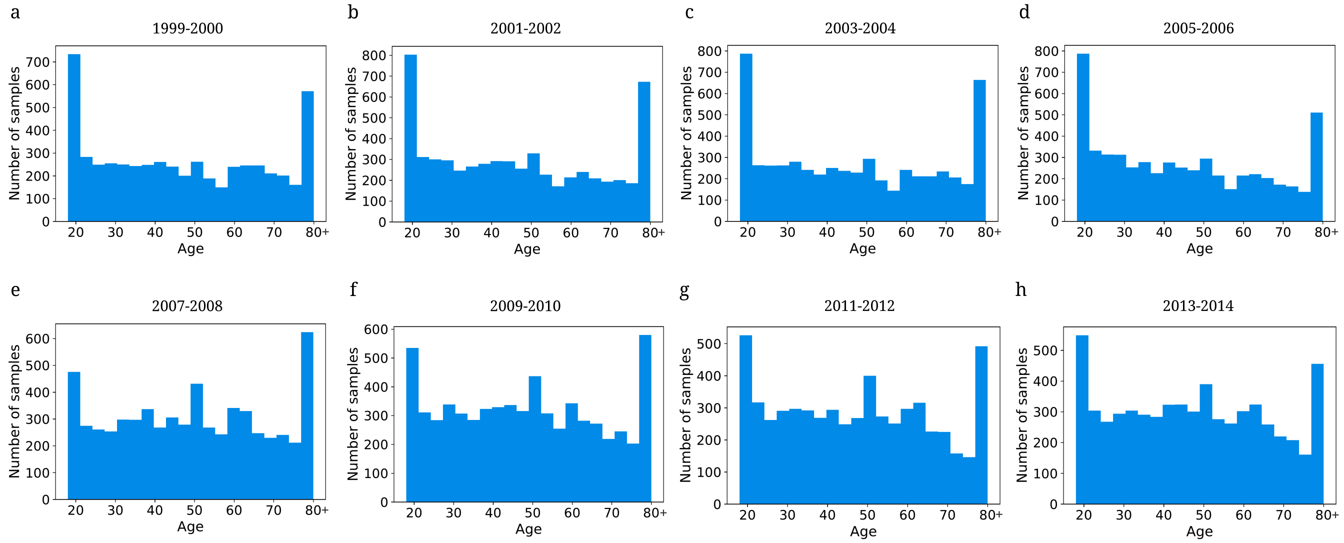

Supplementary Figure 2: (a–h) Histograms of age in different two-year data collection cycles.

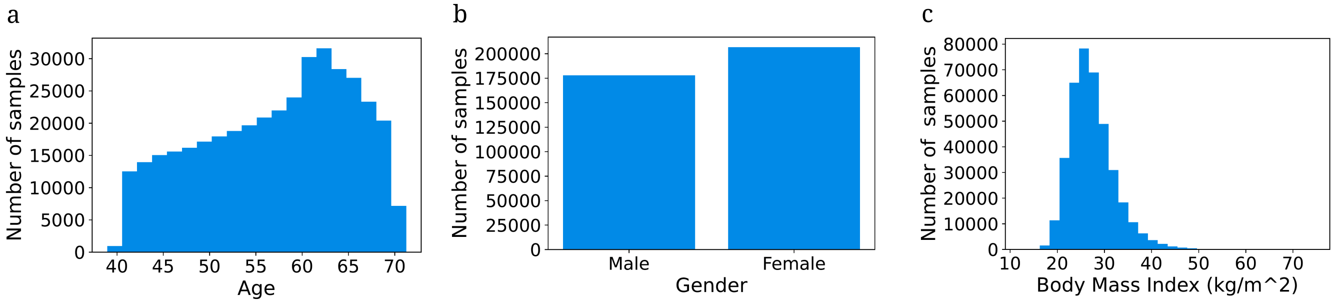

Supplementary Figure 3: (a–c) Histograms of age, gender, and body mass index in the UK Biobank dataset.

Improving prediction power

AUPRC on different mortality prediction tasks

	Logistic Regression		Gradient Boosted Trees		Neural Network
**1-year mortality**	0.1405	***	**0.2307**	***	0.1016
**3-year mortality**	0.3787	***	**0.4397**	***	0.3507
**5-year mortality**	0.5131	***	**0.5464**	***	0.4838
**10-year mortality**	0.7980	***	**0.8212**	***	0.7066
**Age < 40**	0.0441		**0.1047**	*	0.0423
**40 ≤ Age < 65**	0.3436		**0.3823**	***	0.2931
**65 ≤ Age < 80**	0.5263	**	**0.5790**	***	0.4717
**Age ≥ 80**	**0.7447**		0.7071		0.6766

Supplementary Figure 4: The area under the precision-recall curve (AUPRC) of gradient boosted tree models outperforms both linear models and neural networks for seven of our prediction models. $(***)$ represents a p-value < 0.001, $(**)$ represents a p-value < 0.01, and $(*)$ represents a p-value < 0.05. P-values are computed using bootstrap resampling over the tested time points while measuring the difference in area between the curves.

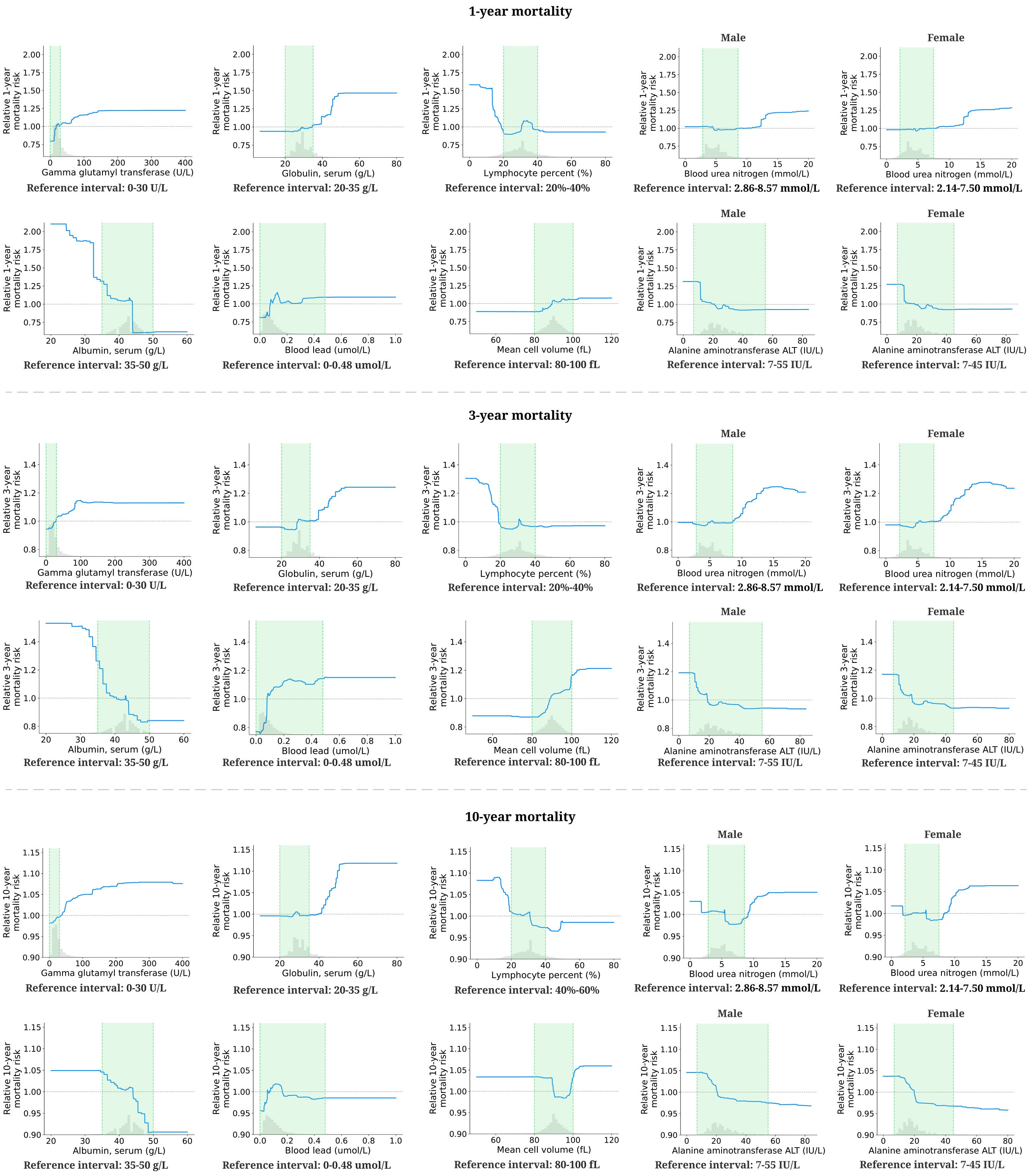

Supplementary Figure 5: **Effect of varying laboratory feature values on 1-, 3- and 10-year mortality risk.** These partial dependence plots show the change in relative 1-, 3- and 10-year mortality risk for all values of a given laboratory feature. The grey histograms on each plot show the distribution of values for that feature in the test set. The green shaded region shows the reference interval of each feature.

External validation of the IMPACT framework on UKB dataset

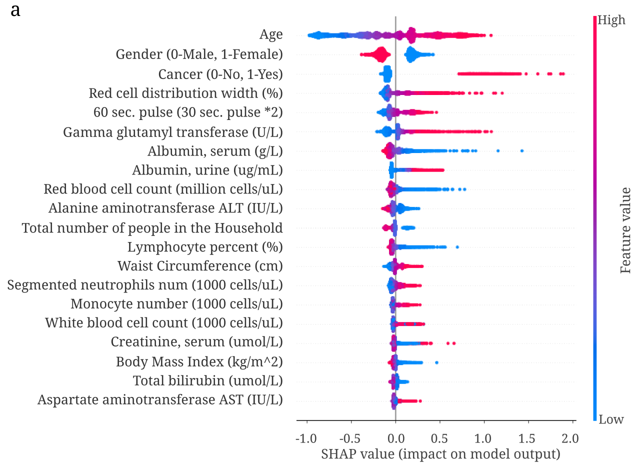

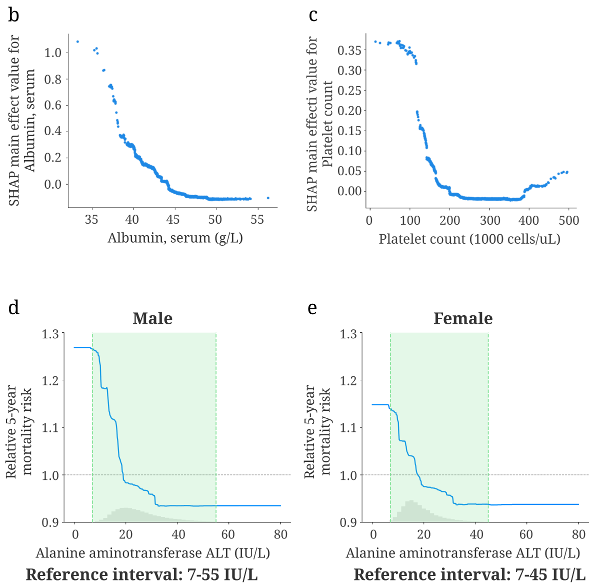

f

Top 20 feature set

	NHANES	Not NHANES	Total
UK Biobank	14	6	20
Not UK Biobank	6	25	31
Total	20	31	51

Supplementary Figure 6: **External validation of the IMPACT framework on UKB dataset.** (a) SHAP summary plot for the 5-year mortality prediction model trained on UKB (51 features) dataset. (b,c) The main effect of serum albumin and platelet count on 5-year mortality of the model trained on UKB (51 features) dataset. (d,e) The relative 5-year mortality risk of alanine aminotransferase ALT on male and female samples of the model trained on UKB (51 features) dataset. (f) The contingency table of the Fisher's exact test that evaluates the significance of the overlap between the top 20 most important overlapping features in the model trained on NHANES (151 features) dataset and the model trained on UKB (51 features) dataset.

External validation of the NHANES mortality prediction model on UKB dataset

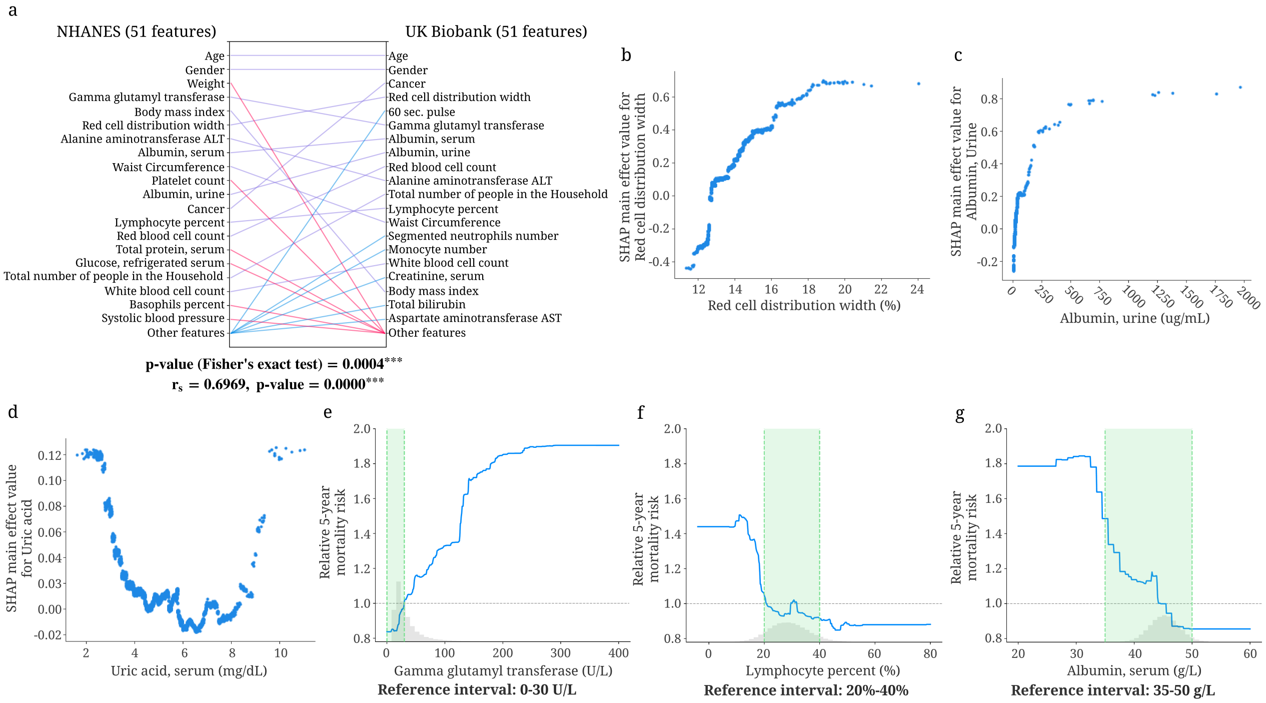

Supplementary Figure 7: **External validation of the NHANES mortality prediction model on the UKB dataset.** (a) Feature importance ranking of models trained on the NHANES (51 features) dataset and the UKB (51 features) dataset. *The SHAP values are calculated using UKB samples.* For each model, the figure shows the 20 most important features of prediction (ordered by importance). The purple line indicates that the feature is in the top 20 features of two models. Blue and red lines indicate the feature is in the top 20 features of one model but not in the top 20 features of the other. The p-value of the Fisher's exact test examines the overlap between the top 20 most important overlapping features in NHANES and UKB models (the contingency table in Supplementary Figure 8G). The Spearman's correlation coefficient is calculated using the feature importance of the overlapping features in NHANES and UKB. $(***)$ represents a p-value < 0.001. (b–d) The main effect of red cell distribution width, urine albumin and serum uric acid on 5-year mortality of the model trained on the NHANES (51 features) dataset and explained using UKB samples. (e–g) The relative 5-year mortality risk of gamma glutamyl transferase, lymphocyte percent and serum albumin of the model trained on the NHANES (51 features) dataset and explained using UKB samples.

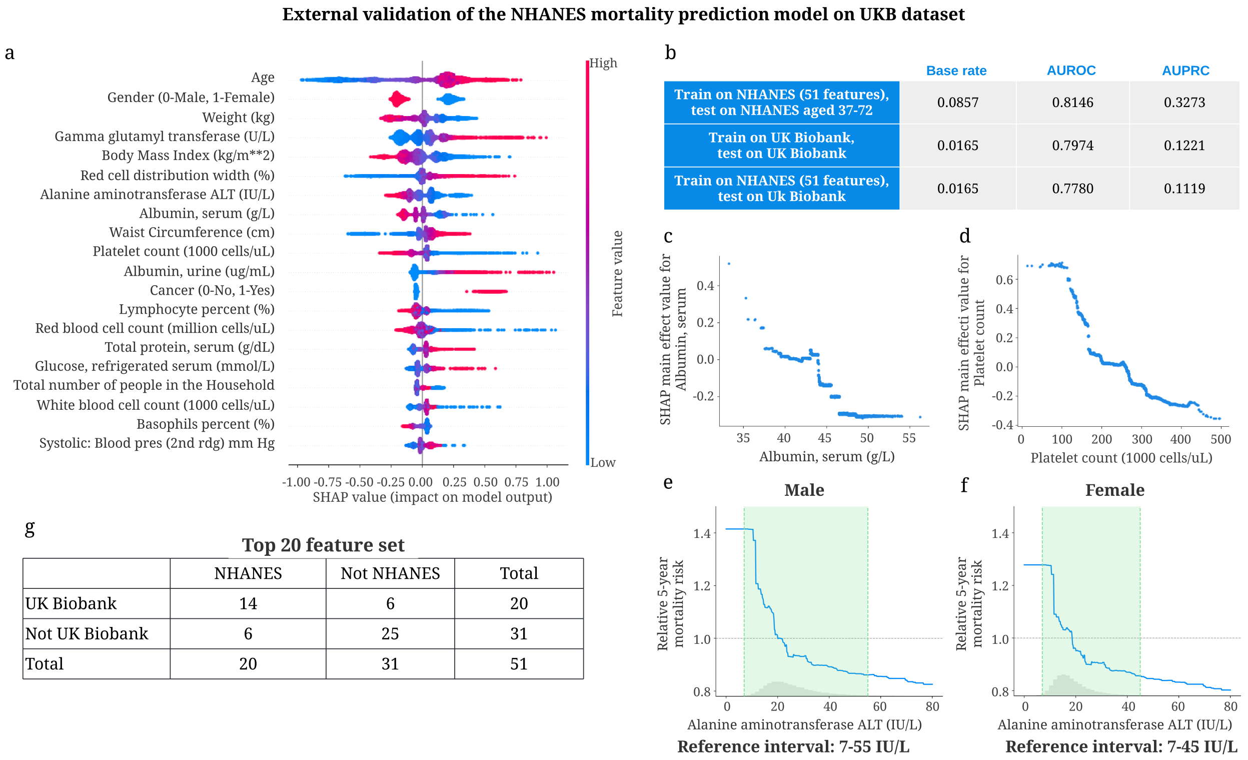

	Base rate	AUROC	AUPRC
Train on NHANES (51 features), test on NHANES aged 37-72	0.0857	0.8146	0.3273
Train on UK Biobank, test on UK Biobank	0.0165	0.7974	0.1221
Train on NHANES (51 features), test on Uk Biobank	0.0165	0.7780	0.1119

	NHANES	Not NHANES	Total
UK Biobank	14	6	20
Not UK Biobank	6	25	31
Total	20	31	51

Supplementary Figure 8: **External validation of the NHANES mortality prediction model on the UKB dataset.** (a) SHAP summary plot for the 5-year mortality prediction model trained on NHANES (51 features) dataset and explained using UKB samples. (b) The predictive performance of the models trained on the NHANES (51 features) and UKB (51 features) datasets. The AUROCs are calculated on the testing set by bootstrapping 1,000 times. (c,d) The main effect of serum albumin and platelet count on 5-year mortality of the model trained on the NHANES (51 features) dataset and explained using UKB samples. (e,f) The relative 5-year mortality risk of alanine aminotransferase ALT on male and female samples of the model trained on the NHANES (51 features) dataset and explained using UKB samples. (g) The contingency table of the Fisher's exact test that evaluates the significance of the overlap between the top 20 most important overlapping features in the model trained on the NHANES (51 features) dataset and the model trained on the UKB (51 features) dataset. Both models are explained using UKB samples.

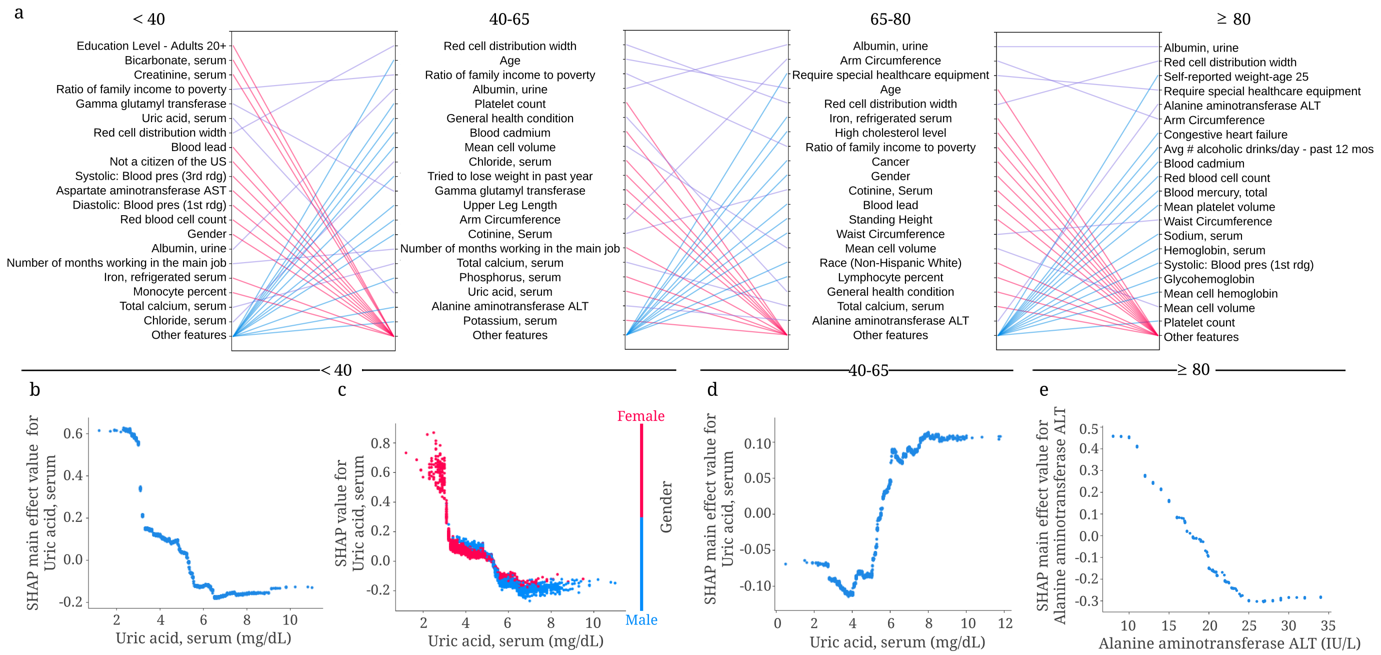

Supplementary Figure 9: **Understanding important risk factors for mortality prediction in different age groups.** (a) Relative importance of input features in <40, 40-65, 65-80 and ≥80 age groups. For each model, the figure shows the 20 most impactful features on prediction (ranked from most to least important). The purple line indicates that the feature is in the top 20 features of two models. Blue and red lines indicate the feature is in the top 20 features of one model, but not in the top 20 features of the other. (b) The main effect of serum uric acid on 5-year mortality in the <40 age group. (c) The SHAP value of serum uric acid in the <40 age group 5-year mortality model. (d) The main effect of serum uric acid on 5-year mortality in the 40-65 age group. (e) The main effect of alanine aminotransferase on 5-year mortality in the ≥80 age group.

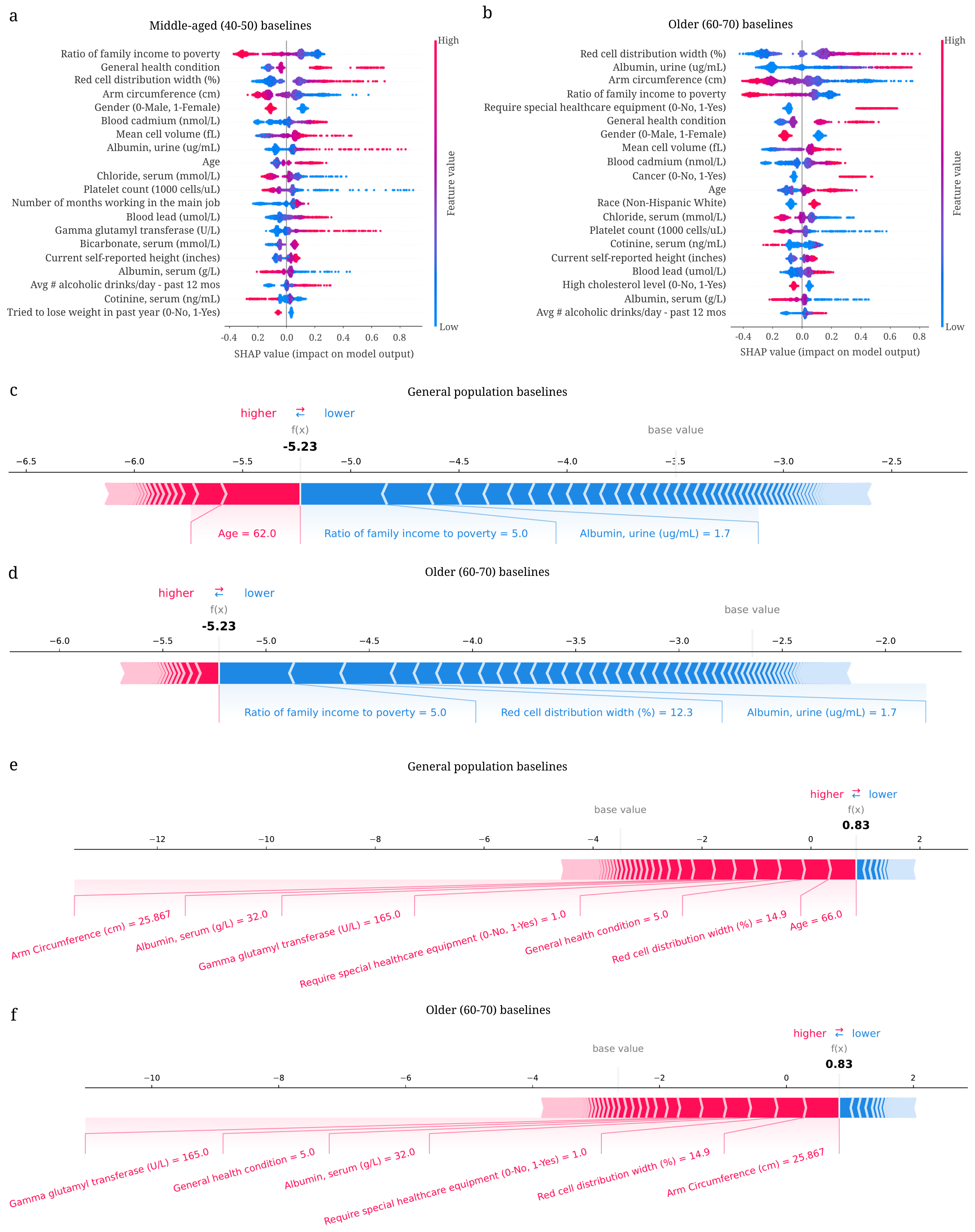

Supplementary Figure 10: **Explaining the 5-year mortality predictions using different baseline distributions.** (a) Explaining the middle-aged subpopulation (40-50 years old) with the baselines of the same age range. (b) Explaining the older subpopulation (60-70 years old) with the baselines of the same age range. (c,d) The individualized explanation for an individual aged 62 using the general population baselines and the older (60-70) baselines. (e,f) the individualized explanation for an individual aged 66 using the general population baselines and the older (60-70) baselines.

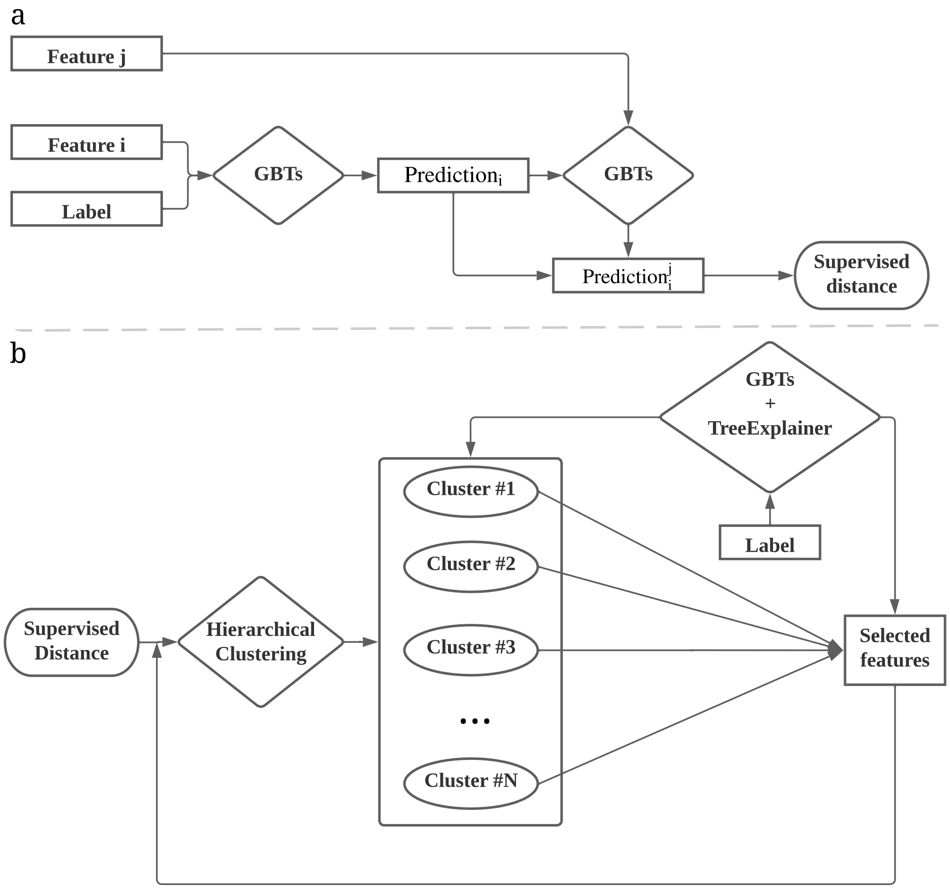

Supplementary Figure 11: (a) The workflow of supervised distance calculation. (b) The workflow of supervised-distance feature selection.

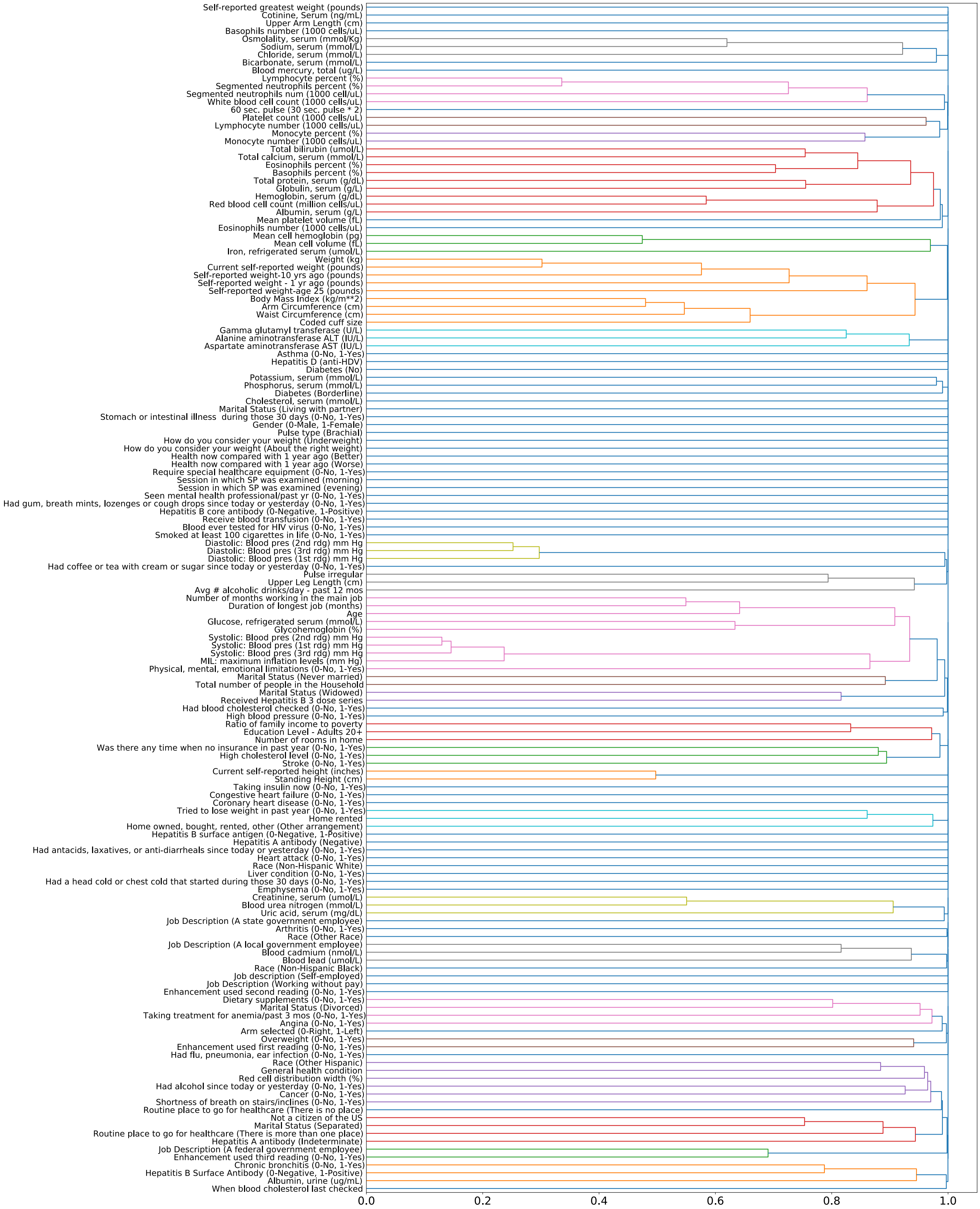

Supplementary Figure 12: The cluster tree of supervised distance based hierarchical clustering. The color threshold is set to 0.98.

a

Follow-up time	Training set (80%) + Testing set (20%)				Temporal validation set			
	Collection cycles	Number of samples	Number of deaths	Base rate	Collection cycles	Number of samples	Number of deaths	Base rate
1-year	1999-2012	41,179	524	1.27%	2013-2014	6,082	53	0.81%
5-year	1999-2008	28,820	2,247	7.80%	2009-2014	7,034	827	11.76%
10-year	1999-2000	5,444	931	17.10%	2001-2014	16,542	4,364	26.38%

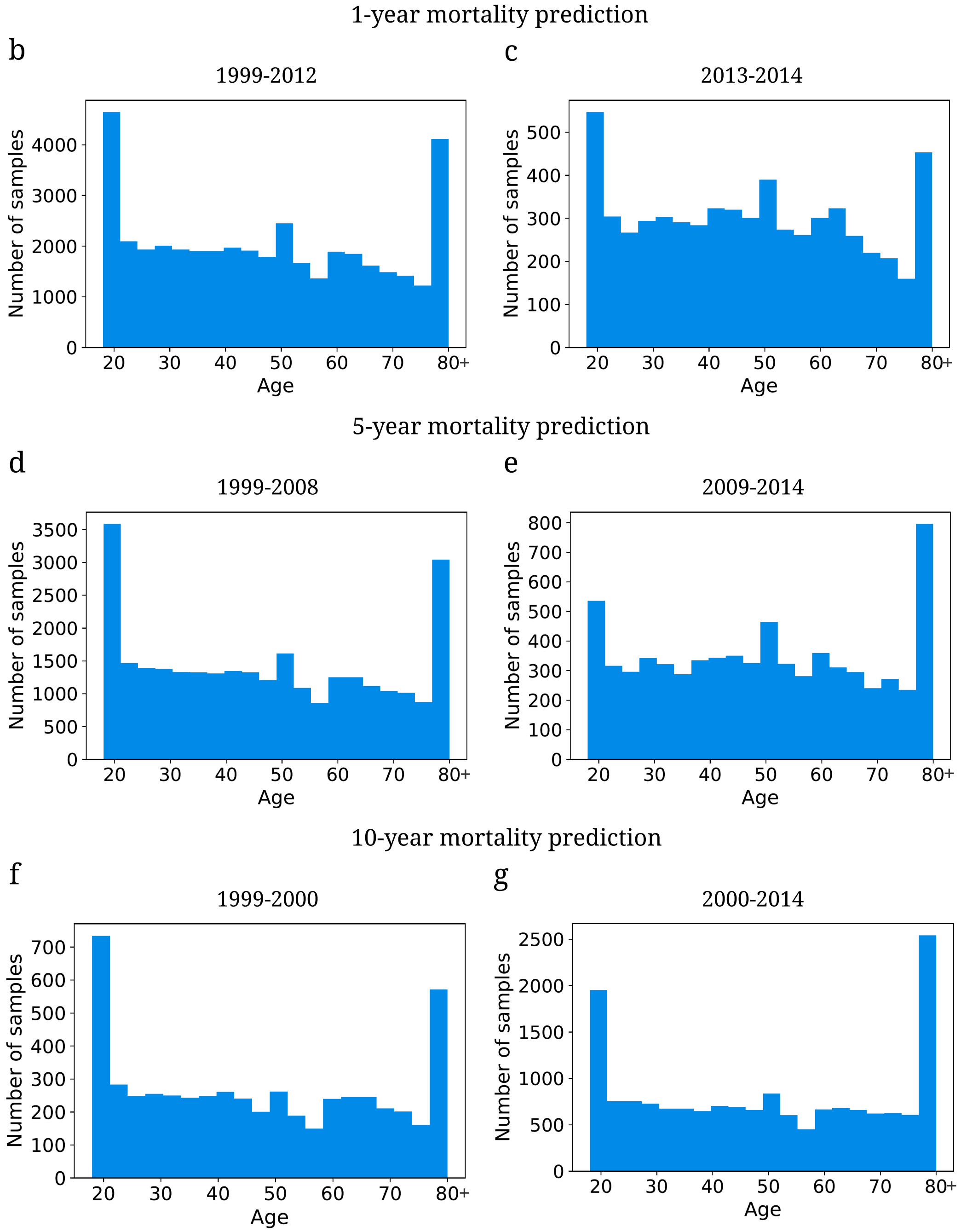

Supplementary Figure 13: (a) Population characteristics of the training/testing and temporal validation sets with different follow-up times. (b–g) Histograms of age in the training/testing set and temporal validation set with different follow-up times.

a

b

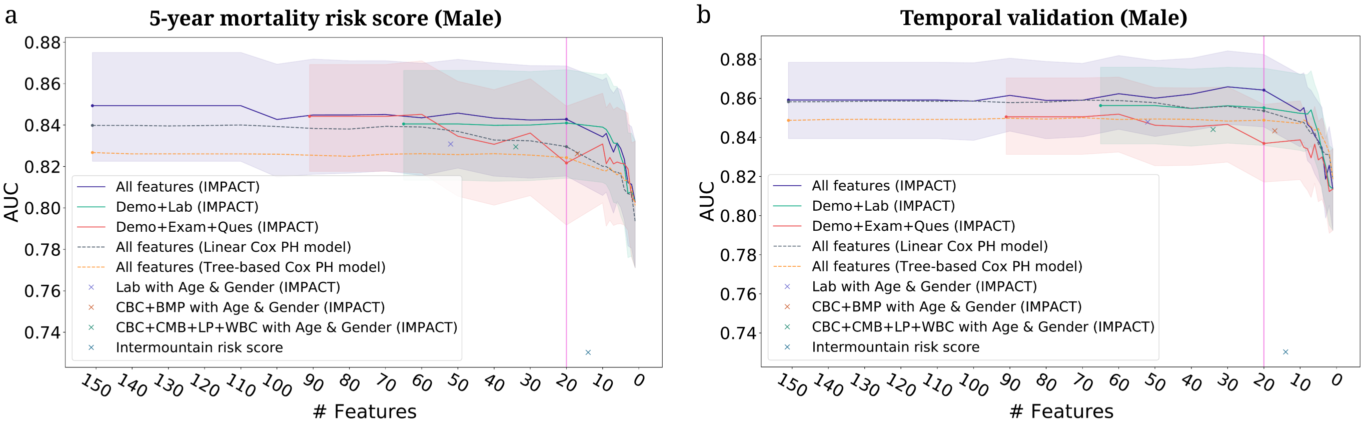

Supplementary Figure 14: (a,b) The AUROC of the models using different feature sets after recursive feature elimination. Lines are mean performance over 1000 random train/test splits, and shaded bands are 95 percent normal confidence intervals. (a) The AUROC of the models tested on the male group in the test set of NHANES 1999-2008. (b) The AUROC of the models testing on the male group in the temporal validation set (NHANES 2009-2014).

Importance Ranking	IMPACT-20	IMPACT-20 (Demo+Lab)
1	Age	Age
2	Albumin, urine (ug/mL)	Blood lead (umol/L)
3	Arm Circumference (cm)	Albumin, urine (ug/mL)
4	Gender (0-Male, 1-Female)	Ratio of family income to poverty
5	Blood lead (umol/L)	Education Level - Adults 20+
6	Ratio of family income to poverty	Red cell distribution width (%)
7	Albumin, serum (g/L)	Chloride, serum (mmol/L)
8	Red cell distribution width (%)	Blood cadmium (nmol/L)
9	Received Hepatitis B 3 dose series	Lymphocyte percent (%)
10	General health condition	Mean cell volume (fL)
11	Mean cell volume (fL)	Red blood cell count (million cells/uL)
12	Number of months working in the main job	Albumin, serum (g/L)
13	Self-reported greatest weight (pounds)	Creatinine, serum (umol/L)
14	Education Level - Adults 20+	Cotinine, Serum (ng/mL)
15	Lymphocyte percent (%)	Platelet count (1000 cells/uL)
16	Require special healthcare equipment (0-No, 1-Yes)	Potassium, serum (mmol/L)
17	Chloride, serum (mmol/L)	Sodium, serum (mmol/L)
18	Blood cadmium (nmol/L)	Alanine aminotransferase ALT (IU/L)
19	Weight (kg)	Blood urea nitrogen (mmol/L)
20	Shortness of breath on stairs/inclines (0-No, 1-Yes)	Race (Non-Hispanic White)
Importance Ranking	IMPACT-20 (Demo+Exam+Ques)	IMPACT (CBC+BMP with age and gender)
1	Age	Age
2	Require special healthcare equipment (0-No, 1-Yes)	Red cell distribution width (%)
3	Arm Circumference (cm)	Mean cell volume (fL)
4	General health condition	Chloride, serum (mmol/L)
5	Education Level - Adults 20+	Gender (0-Male, 1-Female)
6	Gender (0-Male, 1-Female)	Glucose, refrigerated serum (mmol/L)
7	Congestive heart failure (0-No, 1-Yes)	Red blood cell count (million cells/uL)
8	Ratio of family income to poverty	White blood cell count (1000 cells/uL)
9	Diastolic: Blood pres (2nd rdg) mm Hg	Potassium, serum (mmol/L)
10	Systolic: Blood pres (2nd rdg) mm Hg	Creatinine, serum (umol/L)
11	Avg # alcoholic drinks/day - past 12 mos	Platelet count (1000 cells/uL)
12	Cancer (0-No, 1-Yes)	Blood urea nitrogen (mmol/L)
13	Self-reported weight-age 25 (pounds)	Sodium, serum (mmol/L)
14	Number of months working in the main job	Hemoglobin, serum (g/dL)
15	Self-reported greatest weight (pounds)	Mean cell hemoglobin (pg)
16	Duration of longest job (months)	Total calcium, serum (mmol/L)
17	Smoked at least 100 cigarettes in life (0-No, 1-Yes)	Mean platelet volume (fL)
18	Shortness of breath on stairs/inclines (0-No, 1-Yes)	
19	60 sec. pulse (30 sec. pulse * 2)	
20	Current self-reported height (inches)	

Supplementary Table 2: The selected top 20 features of the **5-year** mortality risk scores using different feature types and the features included in CBC and BMP panels.

Importance Ranking	IMPACT-20 (1-year mortality prediction)	IMPACT-20 (10-year mortality prediction)
1	Age	Age
2	Albumin, serum (g/L)	Albumin, urine (ug/mL)
3	Albumin, urine (ug/mL)	Blood lead (umol/L)
4	Lymphocyte percent (%)	General health condition
5	Blood lead (umol/L)	Albumin, serum (g/L)
6	Education Level - Adults 20+	Arm Circumference (cm)
7	Red cell distribution width (%)	Red cell distribution width (%)
8	Cholesterol, serum (mmol/L)	Chloride, serum (mmol/L)
9	Blood mercury, total (ug/L)	Education Level - Adults 20+
10	General health condition	Blood cadmium (nmol/L)
11	Red blood cell count (million cells/uL)	Creatinine, serum (umol/L)
12	Basophils percent (%)	Received Hepatitis B 3 dose series
13	Require special healthcare equipment (0-No, 1-Yes)	Self-reported greatest weight (pounds)
14	Arm Circumference (cm)	Body Mass Index (kg/m**2)
15	Upper Arm Length (cm)	Systolic: Blood pres (2nd rdg) mm Hg
16	Blood cadmium (nmol/L)	Mean cell hemoglobin (pg)
17	Chloride, serum (mmol/L)	Gamma glutamyl transferase (U/L)
18	Avg # alcoholic drinks/day - past 12 mos	Potassium, serum (mmol/L)
19	Systolic: Blood pres (1st rdg) mm Hg	Blood mercury, total (ug/L)
20	Blood urea nitrogen (mmol/L)	How do you consider your weight?

Supplementary Table 3: Selected top 20 features of the 1-year and the 10-year mortality risk scores.

Supplementary References

[1] Tianqi Chen and Carlos Guestrin. "Xgboost: A scalable tree boosting system". In: *Proceedings of the 22nd acm sigkdd international conference on knowledge discovery and data mining*. 2016, pp. 785–794.

[2] Daniel I Feig, Duk-Hee Kang, and Richard J Johnson. "Uric acid and cardiovascular risk". In: *New England Journal of Medicine* 359.17 (2008), pp. 1811–1821. ISSN: 0028-4793.

[3] Earl S Ford et al. "Serum concentrations of uric acid and the metabolic syndrome among US children and adolescents." In: *Circulation* 115.19 (2007), pp. 2526–2532. ISSN: 0009-7322.

[4] Benjamin D Horne et al. "Exceptional mortality prediction by risk scores from common laboratory tests". In: *The American journal of medicine* 122.6 (2009), pp. 550–558.

[5] Seppo Lehto et al. "Serum uric acid is a strong predictor of stroke in patients with non–insulin-dependent diabetes mellitus". In: *Stroke* 29.3 (1998), pp. 635–639. ISSN: 0039-2499.

[6] Scott M Lundberg and Su-In Lee. "A unified approach to interpreting model predictions". In: *Advances in neural information processing systems*. 2017, pp. 4765–4774.

[7] Scott M Lundberg et al. "From local explanations to global understanding with explainable AI for trees". In: *Nature machine intelligence* 2.1 (2020), pp. 2522–5839.

[8] Daniel J Stekhoven and Peter Bühlmann. "MissForest—non-parametric missing value imputation for mixed-type data". In: *Bioinformatics* 28.1 (2012), pp. 112–118.

[9] Blanka Stiburkova et al. "Hereditary renal Hypouricemia type 1 and autosomal dominant polycystic kidney disease". In: *The American journal of the medical sciences* 350.4 (2015), pp. 268–271. ISSN: 0002-9629.

[10] Alexander M Strasak et al. "Serum uric acid and risk of cancer mortality in a large prospective male cohort". In: *Cancer causes & control* 18.9 (2007), pp. 1021–1029. ISSN: 0957-5243.

[11] Minako Wakasugi et al. "Association between hypouricemia and reduced kidney function: a cross-sectional population-based study in Japan". In: *American journal of nephrology* 41.2 (2015), pp. 138–146. ISSN: 0250-8095.
