# Supplementary material for: Interpretable machine learning prediction of all-cause mortality
Source: Commun Med (Lond). 2022 Oct 3;2:125. doi: 10.1038/s43856-022-00180-x (PMC9530124; doi:10.1038/s43856-022-00180-x)
Supplement: Supplementary file 11 — Description of Additional Supplementary Files [file 43856_2022_180_MOESM11_ESM.pdf]

Description of Additional Supplementary Files

File Name: Supplementary Data 1
Description: The lists of the NHANES and UK Biobank features and the features used in all mortality risk scores and feature elimination experiments.

File Name: Supplementary Data 2
Description: The NHANES foreground samples when calculating the SHAP values for the 1-year mortality prediction model.

File Name: Supplementary Data 3
Description: The NHANES foreground samples when calculating the SHAP values for the 3-year mortality prediction model.

File Name: Supplementary Data 4
Description: The NHANES foreground samples when calculating the SHAP values for the 5-year mortality prediction model.

File Name: Supplementary Data 5
Description: The NHANES foreground samples when calculating the SHAP values for the 10-year mortality prediction model.

File Name: Supplementary Data 6
Description: The SHAP values for the NHANES 1-year mortality prediction model.

File Name: Supplementary Data 7
Description: The SHAP values for the NHANES 3-year mortality prediction model.

File Name: Supplementary Data 8
Description: The SHAP values for the NHANES 5-year mortality prediction model.

File Name: Supplementary Data 9
Description: The SHAP values for the NHANES 10-year mortality prediction model.
