# Supplementary material for: Interpretable machine learning prediction of all-cause mortality
Source: Commun Med (Lond). 2022 Oct 3;2:125. doi: 10.1038/s43856-022-00180-x (PMC9530124; doi:10.1038/s43856-022-00180-x)
Supplement: Supplementary file 13 — Reporting Summary [file 43856_2022_180_MOESM13_ESM.pdf]

nature research

Corresponding author(s): Su-In Lee

Last updated by author(s): Aug 24, 2022

Reporting Summary

Nature Research wishes to improve the reproducibility of the work that we publish. This form provides structure for consistency and transparency in reporting. For further information on Nature Research policies, see our Editorial Policies and the Editorial Policy Checklist.

Statistics

For all statistical analyses, confirm that the following items are present in the figure legend, table legend, main text, or Methods section.

n/a	Confirmed	
☐	☒	The exact sample size (n) for each experimental group/condition, given as a discrete number and unit of measurement
☒	☐	A statement on whether measurements were taken from distinct samples or whether the same sample was measured repeatedly
☐	☒	The statistical test(s) used AND whether they are one- or two-sided<br>*Only common tests should be described solely by name; describe more complex techniques in the Methods section.*
☐	☒	A description of all covariates tested
☐	☒	A description of any assumptions or corrections, such as tests of normality and adjustment for multiple comparisons
☐	☒	A full description of the statistical parameters including central tendency (e.g. means) or other basic estimates (e.g. regression coefficient) AND variation (e.g. standard deviation) or associated estimates of uncertainty (e.g. confidence intervals)
☐	☒	For null hypothesis testing, the test statistic (e.g. F, t, r) with confidence intervals, effect sizes, degrees of freedom and P value noted<br>*Give P values as exact values whenever suitable.*
☒	☐	For Bayesian analysis, information on the choice of priors and Markov chain Monte Carlo settings
☐	☒	For hierarchical and complex designs, identification of the appropriate level for tests and full reporting of outcomes
☒	☐	Estimates of effect sizes (e.g. Cohen's d, Pearson's r), indicating how they were calculated

Our web collection on statistics for biologists contains articles on many of the points above.

Software and code

Policy information about availability of computer code

Data collection	No software was used.
Data analysis	All code for our study, including code to train the mortality prediction models and to generate all figures included in the manuscript, are available at https://github.com/suinleelab/IMPACT (archived at https://doi.org/10.5281/zenodo.6899541).<br><br>Our code relies on the following standard Python software packages: numpy, pandas, matplotlib, seaborn.<br>We additionally used the following Python software packages:<br>- missingpy was used for data imputation<br>- Scikit-learn was used for data preprocessing model training<br>- shap was used for model explanation

For manuscripts utilizing custom algorithms or software that are central to the research but not yet described in published literature, software must be made available to editors and reviewers. We strongly encourage code deposition in a community repository (e.g. GitHub). See the Nature Research guidelines for submitting code & software for further information.

Data

Policy information about availability of data

All manuscripts must include a data availability statement. This statement should provide the following information, where applicable:

- Accession codes, unique identifiers, or web links for publicly available datasets
- A list of figures that have associated raw data
- A description of any restrictions on data availability

No new data are generated in this study. The NHANES survey is a public-use data files prepared and disseminated to provide access to the full scope of the data. The

NHANES data for all experiments in the paper is publicly available at https://www.cdc.gov/nchs/nhanes/index.htm. A downloadable version of the dataset is available at https://github.com/suinleelab/IMPACT. The UK Biobank data used in this study is obtained via material transfer agreement as part of Data Access Application 59898. All data is available by UK Biobank via their standard data access procedure (http://www.ukbiobank.ac.uk/register-apply). The data underlying the figures is available in Supplementary Data 2-9.

Field-specific reporting

Please select the one below that is the best fit for your research. If you are not sure, read the appropriate sections before making your selection.

☒ Life sciences ☐ Behavioural & social sciences ☐ Ecological, evolutionary & environmental sciences

For a reference copy of the document with all sections, see nature.com/documents/nr-reporting-summary-flat.pdf

Life sciences study design

All studies must disclose on these points even when the disclosure is negative.

Sample size	Each year NHANES examines a nationally representative sample of roughly 5,000 individuals across the Unites States. In this study, we includes 47,261 samples with known mortality status who participated in NHANES 1999-2014.
Data exclusions	For NHANES dataset, we exclude variables that are missing for more than 50% of the participants and highly correlated features with correlations greater than 0.98 for NHANES dataset.
Replication	We used a held-out test set with 20% of samples that were not used during training, implying strong statistical significance. Furthermore, we ensure generalizability by performing temporal validation of the risk scores and external validation of feature importances and important relationships with the UK Biobank dataset.
Randomization	The training and validation sets were randomly split.
Blinding	Preprocessing of datasets was blinded to labels. Models were trained to predict mortality and thus labels were used for training.

Reporting for specific materials, systems and methods

We require information from authors about some types of materials, experimental systems and methods used in many studies. Here, indicate whether each material, system or method listed is relevant to your study. If you are not sure if a list item applies to your research, read the appropriate section before selecting a response.

Materials & experimental systems

n/a	Involved in the study	
☒	☐	Antibodies
☒	☐	Eukaryotic cell lines
☒	☐	Palaeontology and archaeology
☒	☐	Animals and other organisms
☒	☐	Human research participants
☒	☐	Clinical data
☒	☐	Dual use research of concern

Methods

n/a	Involved in the study	
☒	☐	ChIP-seq
☒	☐	Flow cytometry
☒	☐	MRI-based neuroimaging
